# The impact of powerful authorities and trustful taxpayers: evidence for the extended slippery slope framework from Austria, Finland, and Hungary

**DOI:** 10.1080/01442872.2019.1577375

**Published:** 2019-02-11

**Authors:** Katharina Gangl, Eva Hofmann, Barbara Hartl, Mihály Berkics

**Affiliations:** a Department of Economic and Social Psychology, University Goettingen, Göttingen, Germany; b Competence Center of Empirical Research Methods, WU Vienna University of Economics and Business, Vienna, Austria; c Institute of Organization, Johannes Kepler University Linz, Vienna, Austria; d Institute of Psychology, ELTE Eötvös Loránd University, Budapest, Hungary

**Keywords:** Tax compliance, tax evasion, trust, relationship climate

## Abstract

Tax authorities utilize a wide range of instruments to motivate honest taxpaying ranging from strict audits to fair procedures or personalized support, differing from country to country. However, little is known about how these different instruments and taxpayers’ trust influence the generation of interaction climates between tax authorities and taxpayers, motivations to comply, and particularly, tax compliance. The present research examines the extended slippery slope framework (eSSF), which distinguishes tax authorities’ instruments into different qualities of power of authority (coercive and legitimate) and trust in authorities (reason-based and implicit), to shed light on the effect of differences between power and trust. We test eSSF assumptions with survey data from taxpayers from three culturally different countries (*N* = 700) who also vary concerning their perceptions of power, trust, interaction climates, and tax motivations. Results support assumptions of the eSSF. Across all countries, the relation of coercive power and tax compliance was mediated by implicit trust. The connection from legitimate power to tax compliance is partially mediated by reason-based trust. The relationship between implicit trust and tax compliance is mediated by a confidence climate and committed cooperation. Theoretical and practical implications are discussed.

## Introduction

Tax authorities apply different measures to increase tax compliance. These measures differ from country to country. Nevertheless, many practical and theoretical accounts such as the slippery slope framework of tax compliance (Kirchler 2007) have categorized different measures into two basics approaches: the power approach and the trust approach (Feld and Frey 2007; Kirchler 2007; Luttmer and Singhal 2014). The power approach (also termed deterrence or the command and control approach) relies on frequent audits and severe fines in case of tax evasion (Allingham and Sandmo 1972). In contrast, the trust approach originates from transparency, fair procedures, or the conviction that paying taxes honestly is a binding social norm (Braithwaite 2003a; Kirchler, Hoelzl, and Wahl 2008; Luttmer and Singhal 2014). The mutual influence, interaction, and dynamic between these two approaches is seen as important for tax compliance. Whereas some authors suggested that power and trust mutually enhance each other’s effect on compliance, others assumed that power can also erode trust and in turn could reduce compliance (Bijlsma-Frankema and Costa 2005; Das and Teng 1998). However, only few studies exist that examined this dynamic empirically (Hofmann et al. 2017).

To explain the dynamic between power and trust, the original slippery slope framework was extended by differentiating the power of tax authorities into coercive and legitimate power and trust in tax authorities into reason-based and implicit trust (Gangl, Hofmann, and Kirchler 2015). Based on this, the extended slippery slope framework (eSSF) describes how the different qualities of power and trust interact with each other and lead to specific relationship climates between tax authorities and taxpayers and to tax motivations that determine tax payments (Gangl, Hofmann, and Kirchler 2015). Coercive power is suggested to decrease implicit trust in tax authorities and to lead to an antagonistic climate and an enforced motivation to pay taxes. Legitimate power fosters reason-based trust, a service climate, and a voluntary motivation to pay taxes. Based on positive experiences with tax authorities, the eSSF suggests that over time, reason-based trust evolves to implicit trust, which leads to a confidence climate with a committed motivation to pay taxes (Gangl, Hofmann, and Kirchler 2015). Whereas the original slippery slope framework received empirical support in numerous survey studies and experiments (e.g. Muehlbacher, Kirchler, and Schwarzenberger 2011; Wahl, Kastlunger, and Kirchler 2010), only some of the assumptions of the eSSF were tested, mostly based on experiments (e.g. Hartl et al. 2015; Hofmann et al. 2014). Throughout empirical analyses of all dynamics between power and trust that are assumed in the eSSF and studies based on data from real taxpayers living in different countries are rare. Closing this empirical gap allows an examination of the eSSF and tests whether tax authorities’ approaches directly or indirectly influence tax compliance via changing the perceived relationship with authorities and taxpayers’ motivations. Testing eSSF assumptions with data from different countries also informs whether different approaches aimed to increase tax compliance work in the same manner independent of the country and cultural specifics in which they are applied.

The aim of the present paper is to test the eSSF assumption on the psychological processes and consequences of the power-trust dynamic. Therefore, the present paper examines the underlying psychological processes that allow power and trust approaches to influence tax compliance. To increase the generalizability of found results and to test whether tax authorities’ approaches have similar consequences in different countries, we also aim to analyse taxpayer data from different countries that have varying tax cultures.

### The extended slippery slope framework

Tax researchers agree that tax authorities need to apply the full range of possible instruments in order to guarantee tax compliance from citizens (Alm and Torgler 2011; Braithwaite 2003a, 2003b). These instruments include the classical deterrence approach, based on the force of the law through coercive audits and fines, and alternative approaches including regulation, incentives, participation, fairness, or support and service. Although the positive effect of each of these approaches on tax compliance received much theoretical and empirical support (Alm and Torgler 2011; Blackwell 2007; Murphy 2004; Wahl, Kastlunger, and Kirchler 2010), their dynamic and joint influence on tax compliance is still largely unexplored (Gobena and Van Dijke 2016).

Experiments from fields other than tax compliance indicated that coercive control and punishment is more effective if applied in a fair versus unfair manner (Mooijman et al. 2017; Verboon and van Dijke 2011). Similar experiments demonstrated that punishments exerted by a trusted authority have stronger effects on moral judgments about rule-breaking behaviour than punishments exerted by an untrusted authority (Mulder, Verboon, and De Cremer 2009). A meta-analysis also showed that punishments are more effective in countries in which general trust is high compared to low (Balliet, Mulder, and van Lange 2013). On the other hand, it is claimed that coercive control and punishment break the social contract between authorities and citizens, decreasing trust between authorities and fellow citizens (Feld and Frey 2007; Kramer 1999; Tenbrunsel and Messick 1999); this in turn can lead to lower cooperation (Ariel 2012; Gangl et al. 2014; Slemrod, Blumenthal, and Christian 2001). Thus, coercive punishment, trust, and fair procedures can mutually work together to either strengthen or weaken each other and in turn have different effects on cooperation with authorities. However, thorough theoretical and empirical examinations of the power-trust dynamic, particularly in tax research, is missing. For instance, it is not clear how audits and fines may increase trust in the tax system instead of decreasing it. Insights into these mechanisms could fortify our understanding of contradictory results from studies that have shown a positive effect (Hasseldine et al. 2007), no effect (Ariel 2012), or even negative effects of audits and fines (Gangl et al. 2014; Slemrod, Blumenthal, and Christian 2001).

The eSSF aims to explain these contradictory findings and the dynamics and interactions between different approaches. Therefore, the eSSF categorizes tax authorities’ different approaches into various qualities of power (i.e. coercive power and legitimate power) and different qualities of trust (i.e. reason-based trust and implicit trust).

The power of tax authorities, which is defined based on the theory of the “bases of social power” (French and Raven 1959; Pierro et al. 2012), is differentiated into coercive power and legitimate power (Raven, Schwarzwald, and Koslowsky 1998). These two qualities of power are conceptualized as independent qualities; they can be applied alone or in combination with each other.

Coercive power represents the power to punish and the power to reward; thus, it becomes either a negative and positive incentive for behaviour. Examples of punishment are fines or negative disclosure through transparent tax returns (Bø, Slemrod, and Thoresen 2014) or black lists (Perez-Truglia and Troiano 2015). Examples of rewards are wellness vouchers for timely payment (Koessler et al. 2016) or the promise of privileged treatment (Simone, Sansing, and Seidman 2013 ). Both punishments and rewards likely crowd out intrinsic motivation (Deci 1971) and are seen as a form of coercion (Raven, Schwarzwald, and Koslowsky 1998).

Legitimate power is defined as the perception that authorities work based on a legitimate foundation, expertise and information provision, and a positive reputation (Raven, Schwarzwald, and Koslowsky 1998). Different subcategories of legitimate power are related to transparency and fairness (Wenzel 2002), legitimate regulation (Murphy 2005), taxpayer’s voice and participation (Pommerehne and Weck-Hannemann 1996), the provision of relevant information (Alm et al. 2010), and to supportive services (Gangl et al. 2013).

Based on the socio-cognitive trust theory (Castelfranchi and Falcone 2010), trust is differentiated into reason-based and implicit trust. Reason-based trust is defined as the deliberate decision to trust tax authorities based on their perceived goals; their perceived competence, motivation, and benevolence; and perceived supportive external circumstances. Reason-based trust is related to tax knowledge (Eriksen and Fallan 1996), the perceived competence and good intentions of authorities (e.g. Gangl et al. 2013; Murphy 2004), and perceived institutional quality and corruption (e.g. Cummings et al. 2009).

In contrast, implicit trust is an automatic and associative reaction to a stimuli such as a friendly face or voice or official-looking documents (Castelfranchi and Falcone 2010). However, reason-based and implicit trust are related. Along System 1 and System 2 conceptualizations, it is assumed that trust is first based on rational considerations and becomes implicit over time as a result of positive experiences (Evans 2008). Implicit trust summarizes determinants of tax compliance related to marketing and newspaper campaigns (Cyan, Koumpias, and Martinez-Vazquez 2017), social norms of tax honesty (Hallsworth et al. 2017), and the perception of a shared identity (i.e. patriotism; Gangl, Torgler, and Kirchler 2016). Nudges, such as automatic reminders or prepopulated tax forms (Behavioural Insights Team 2011; Chirico et al. 2017), are also likely to trigger automatic and implicit trust.

**Figure 1.**
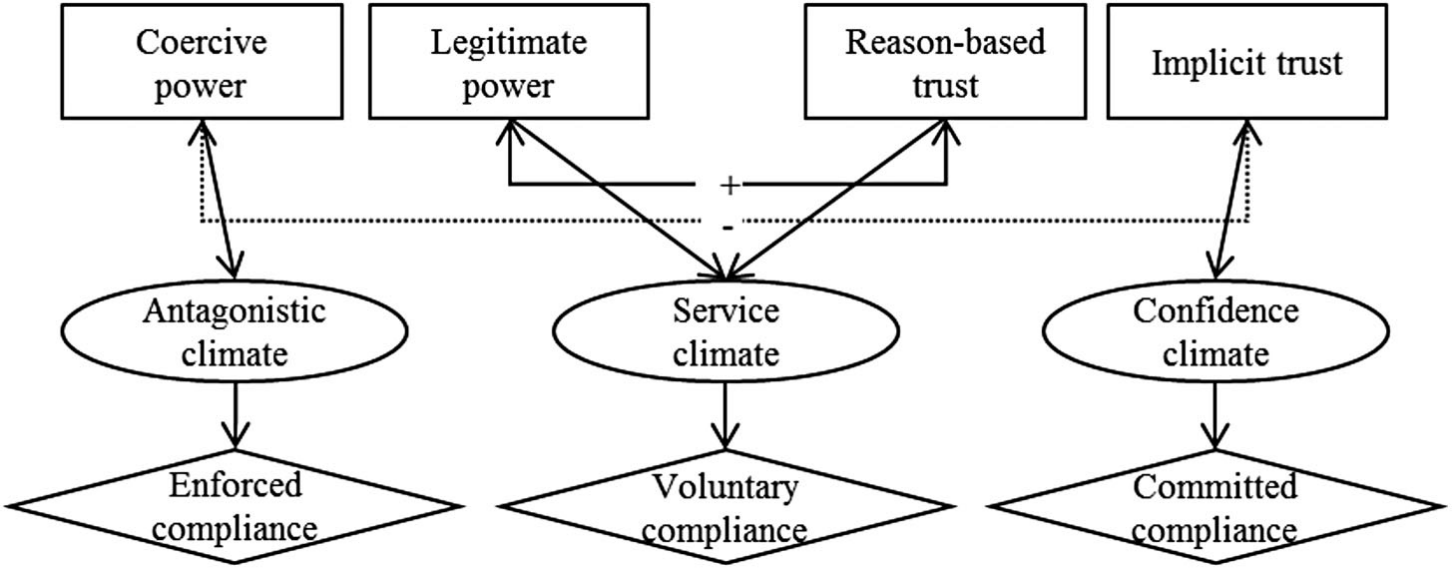
Extended slippery slope framework (Gangl, Hofmann, and Kirchler 2015).

The eSSF assumes that two main mechanisms drive the dynamic between the different qualities of power and trust that in turn impact the perceived relationship climate between authorities and taxpayers and taxpayers’ motivation to comply (Figure 1; Gangl, Hofmann, and Kirchler 2015). First, the eSSF suggests a negative relationship between coercive power and implicit trust, which leads either to an antagonistic or confidence-based climate. In an antagonistic climate, coercive power is high and implicit trust is low, and tax authorities are perceived to persecute taxpayers primarily interested in catching them as tax evaders. As a consequence, taxpayers pay their taxes because they feel enforced to do so by control and punishment (Braithwaite 2003a, 2003b; Feld and Frey 2002). In a confidence climate, implicit trust is high whereas coercive power is low. The interaction between tax authorities and taxpayers is characterized by mutual trust and respect; therefore, harsh coercive power is not perceived as necessary. In such a climate, taxpayers feel committed to the tax system and see taxpaying as their moral obligation.

Second, the eSSF suggests a positive relationship between legitimate power and reason-based trust that fosters a service climate. In a service climate, tax authorities and taxpayers have a professional, bureaucratic relationship in which tax authorities as service providers interact with taxpayers as clients (Alm and Torgler 2011; Braithwaite 2003b).

Based on these assumptions, it can be suggested that a pure coercive deterrence approach has a negative effect on trust, interaction climates, and motivation, and in turn tax compliance intentions. In contrast, if authorities’ power is perceived to be legitimate, or if authorities’ coercive power is combined with legitimate power, a positive effect on trust, climate, motivation, and tax compliance can be expected.

Experiments on the effects of coercive power and legitimate power, applied solely or in combination, largely confirmed the above mentioned assumptions (Gangl et al. 2017; Hartl et al. 2015; Hofmann et al. 2014, 2017). In these experiments, participants were asked to act as self-employed taxpayers who must pay taxes on a given income. Low versus high coercive and legitimate power was manipulated by describing tax authorities as applying lenient or severe controls (to manipulate coercive power) with ill or well-trained tax officers (to manipulate legitimate power). These experiments showed, as expected, that coercive power but not legitimate power increases taxpayers’ reactance and reduces implicit trust in tax authorities, which in turn leads to an antagonistic climate and enforced motivation to comply (Gangl et al. 2017; Hofmann et al. 2014). However, these experiments only analysed the effect of coercive and legitimate power but not trust (largely due to the difficulty of experimentally manipulating implicit trust). In addition, survey studies on representative samples of self-employed taxpayers from Austria and the Netherlands on the correlation of different motivations to comply (i.e. enforced compliance, voluntary cooperation, and committed cooperation with tax compliance intentions) were conducted (Gangl et al. 2013). These studies showed that enforced compliance was negatively related to tax compliance, whereas voluntary and committed cooperation were positively correlated to tax compliance. Thorough survey studies that analysed all assumed dynamics between power and trust in the eSSF are rare. One exception offered confirmation of the framework (Gangl et al. 2016). However, this study was small in sample size, based on just one country, and did not include tax compliance intentions. Hence, it is still not clear how robust the assumed dynamics are between power, trust, interaction climates, motivation to comply, and tax compliance.

### National differences in tax behaviour

In the present study, we used data from three European countries that strongly vary concerning authorities’ perceived power and citizens’ trust in authorities: Austria, Finland, and Hungary. According to the European Value Survey (2011), only 15.7% of citizens in Austria and 19.1% in Finland claim that the state should increase control of firms. In contrast, 43.8% of Hungarians demand more frequent and efficient control. This might indicate that in Hungary there are higher authoritarian attitudes and that authority is perceived as more legitimate than in Finland or Austria. Particularly, trust in authorities differs across the three countries (e.g. Kogler et al. 2013). Data from the European Value Survey (2011) showed that citizens’ trust in national authorities (i.e. the parliament) is highest in Finland (42.1% of the Finnish have confidence in parliament), followed by Austria (29.9%) and Hungary (20.7%). The data from the European Value Survey indicated that Finland is the democratic extreme, whereas Hungary is on the authoritarian extreme and Austria represents a middle position between the two.

**Figure 2.**
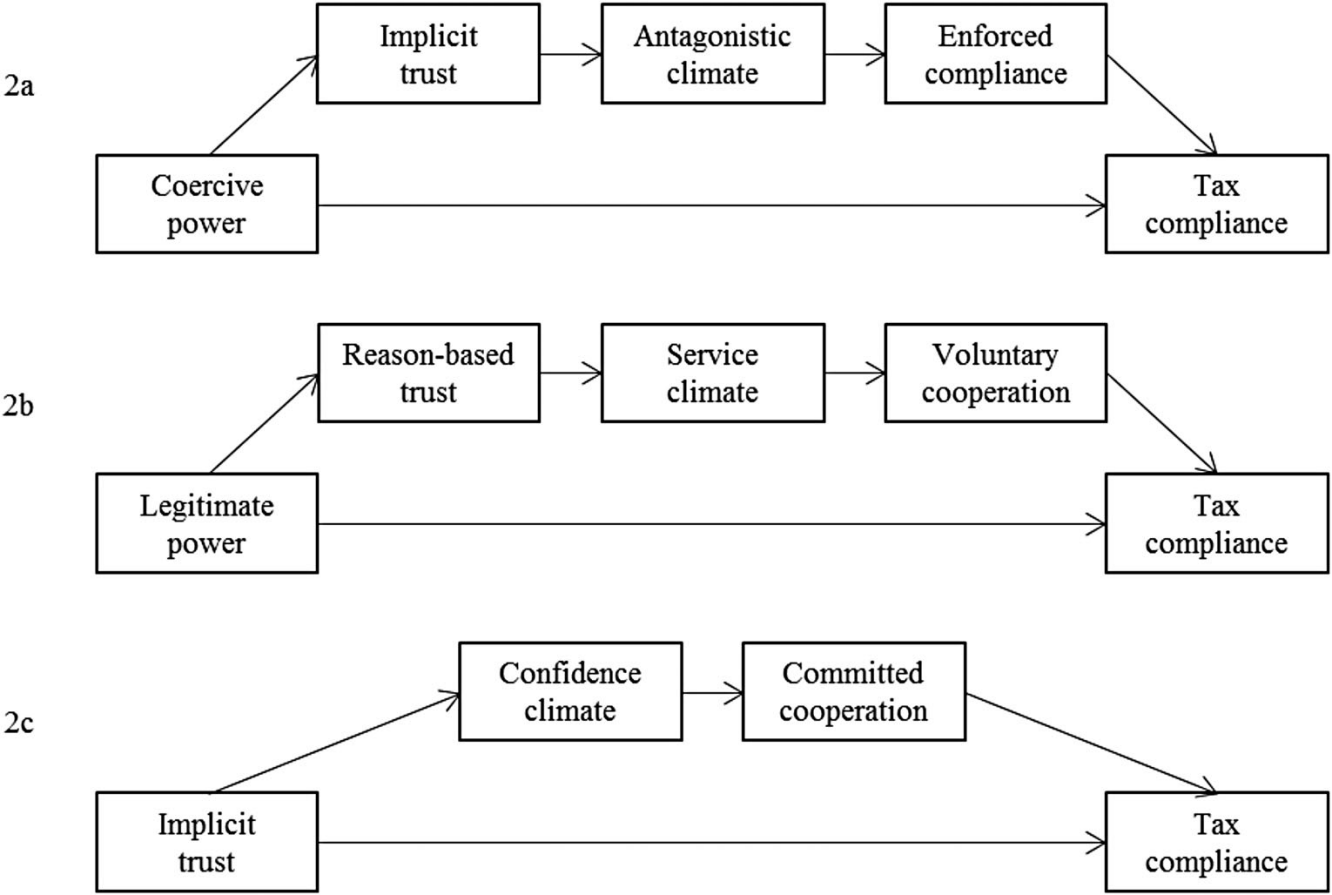
(a – c) The extended slippery slope framework and tax compliance.

The aim of the present study is to test the eSSF assumptions by examining whether the relationship between power, trust, and tax compliance intentions is mediated by perceived tax climates and motivations. Figure 2 visualizes our hypotheses. To increase the robustness of our results, we examined the data of taxpayers from three countries differing in regulation and citizens’ trust – Austria, Finland, and Hungary – who likely also differ concerning power and trust, perceived tax climates, and motivation to comply with taxes.

## Method

### Samples and procedure

Overall, 700 taxpayers (253 Austrians, 223 Finnish, and 224 Hungarians) completed an online survey. The first item on the questionnaire was a filter item asking whether participants had ever paid taxes in the past. Only those participants who indicated that they had experience with taxpaying continued the survey. After additional exclusion of participants who denied the question, “I have read all written instructions and questions carefully and have given my personal opinion”, data of 249 Austrian (57% male; *M*_age_ = 35.06, *SD* = 12.82), 219 Finnish (48% male; *M*_age_ = 45.94, *SD* = 13.34), and 222 Hungarian participants (39% male; *M*_age_ = 39.98, *SD* = 12.50) were considered in the analyses. Participants in Austria and Hungary were recruited from acquaintances of university members. Finnish participants were recruited by a market research agency and received a choice of low-price items as a reward for participation.

### Material

The online questionnaire was used to assess (a) *perceived coercive power* (three items; e.g. “Tax authorities punishes severely”), (b) *perceived legitimate* power (seven items; e.g. “Tax authorities do share understandable information”)*,* (c) *implicit trust* (three items; e.g. “I trust tax authorities most of the time automatically”), (d) *reason-based trust* (14 items; e.g. “I trust tax authorities because they have committed employees”), (e) *perceived antagonistic climate* (three items; e.g. “Between the tax authority and taxpayer there exists a climate of inconsiderateness”), (f) *perceived service climate* (three items; e.g. “The relationship between the tax authority and taxpayer is service-oriented in nature”), (g) *perceived confidence climate* (three items; e.g. “The relationship between the tax authority and taxpayer is characterized by joint responsibility”), (h) *enforced compliance* (four items; e.g. “When I pay taxes, I do so because I know I will be audited”), (i) *voluntary cooperation* (four items; e.g. “When I pay taxes, I do so because the tax authority will probably reciprocate my cooperation”), and (j) *committed cooperation* (four items; e.g. “When I pay taxes, I do so because I feel a moral obligation to pay taxes”). Participants were also surveyed on tax compliance (one item; “How likely will you pay your taxes for the current year correctly and in full extent to the tax authority?”) and socio-demographic characteristics. Scales and items are based on Hofmann et al. (2017) and Gangl et al. (2015) and were answered on a 7-point Likert scale (1 = *totally disagree*, 7 = *totally agree*). Cronbach’s α for all scales were sufficient (see Table 1).

**Table 1.** Reliabilities, means, standard deviations, and partial inter-correlations of the slippery slope framework scales for Austria, Finland, and Hungary.

	α	AUT M (SD)	FIN M (SD)	HUN M (SD)	1	2	3	4	5	6	7	8	9	10
1 Coercive Power	.75	4.60 (1.41)	4.58 (1.38)	5.12 (1.22)										
2 Legitimate Power	.81	3.85 (0.91)	4.27 (0.96)	3.65 (0.84)	−.12**									
3 Implicit Trust	.93	3.84 (1.88)	4.40 (1.65)	3.72 (1.65)	−.13**	.54***								
4 Reason-based Trust	.91	3.36 (1.04)	3.98 (1.16)	3.48 (1.21)	−.15***	.66***	.73***							
5 Antagonistic Climate	.90	3.94 (1.61)	3.72 (1.67)	4.44 (1.72)	.42***	−.33***	−.35***	−.35***						
6 Service Climate	.86	3.14 (1.41)	4.16 (1.49)	2.76 (1.35)	−.25***	.57***	.43***	.58***	−.38***					
7 Confidence Climate	.88	3.05 (1.39)	3.64 (1.48)	2.62 (1.30)	−.22***	.62***	.56***	.70***	−.44***	.68***				
8 Enforced Compliance	.84	4.11 (1.47)	3.75 (1.54)	3.67 (1.69)	.23***	.03	−.07	−.04	.31***	−.01	−.08*			
9 Voluntary Cooperation	.75	3.34 (1.30)	3.80 (1.34)	2.89 (1.33)	−.06	.52***	.41***	.53***	−.20***	.48***	.51***	.17***		
10 Committed Cooperation	.90	4.90 (1.56)	5.49 (1.32)	5.50 (1.42)	−.07	.36***	.35***	.41***	−.17***	.23***	.26***	−.16***	.26***	
11 Intended tax behaviour		6.13 (1.54)	6.34 (1.15)	6.28 (1.23)	.02	.11**	.15***	.15***	−.11**	.07	.08*	−.09*	.07	.27***

Note. α = Cronbach-α, AUT = Austria, FIN = Finland, HUN = Hungary.

*p < .05, **p < .01, ***p < .001.

## Results

In the following section, our preliminary analysis aimed to show that Austria, Finland, and Hungary significantly differ regarding perceived power, taxpayer trust, interaction climates, and motivation for tax compliance. After that, we tested the dynamic between power and trust, interaction climates, motivation, and tax compliance intentions by applying a mediation analysis.

### Preliminary analysis: differences between Austrian, Finish, and Hungarian taxpayers

To confirm that Austria, Finland, and Hungary differ concerning power and trust, perceived tax climates, and motivations to comply with taxes, ANOVAs (Table 1) were computed. Coercive power was perceived as higher in Hungary than in Austria and Finland (*F*(687, 2) = 11.60, *p* < .001, *η_p_^2^* = .03), whereas legitimate power was significantly perceived as higher in Finland than in Austria and Hungary (*F*(687, 2) = 27.03, *p* < .001, *η_p_^2^* = .07). Trust – both implicit (*F*(687, 2) = 9.83, *p* < .001, *η_p_^2^* = .03) and reason-based (*F*(687, 2) = 19.24, *p* < .001, *η_p_^2^* = .05) – was higher in Finland than in Austria and Hungary. An antagonistic climate was perceived as higher in Hungary than in Austria and Finland (*F*(687, 2) = 11.02, *p* < .001, *η_p_^2^* = .03). A service (*F*(687, 2) = 57.82, *p* < .001, *η_p_^2^* = .14) and confidence climate (*F*(687, 2) = 29.87, *p* < .001, *η_p_^2^* = .08) were both perceived as highest in Finland, as second highest in Austria, and as lowest in Hungary. Finish and Hungarian participants appeared to feel less enforced to pay taxes than Austrians (*F*(687, 2) = 5.25, *p* < .01, *η_p_^2^* = .02). Finnish participants were the highest motivated to pay voluntary; Austrians were second highest; and Hungarians were the least motivated to pay their taxes voluntarily (*F*(687, 2) = 26.24, *p* < .001, *η_p_^2^* = .07). Austrians felt less committed to pay their taxes than Finnish and Hungarian participants (*F*(687, 2) = 13.55, *p* < .001, *η_p_^2^* = .04). However, concerning intended tax behaviour, no difference was observed between the three countries (*F*(687, 2) = 1.63, *p* = .20, *η_p_^2^* = .01). Table 1 shows means and standard deviations separately for Austria, Finland, and Hungary.

### Test of the extended slippery slope framework of tax compliance

To test whether the assumed dynamics between power and trust were related to tax compliance intention via perceived climates and motivations, we conduced serial mediation analyses (Process Model 6; Hayes 2012, 2013), controlling for participants’ country. The first analysis comprised coercive power as a predictor and tax compliance as criterion, whereby implicit trust, antagonistic climate, and enforced compliance were the mediators (Figure 2(a)). Results showed a full mediation (95% CI [-0.0985; -0.0300], R2 =.04). However, coercive power was only related to implicit trust (*b* = -.15, *SE* = .05, *p* = .002), which in turn influenced tax compliance intentions (*b* = .12, *SE* = .03, *p* < .001; 95% CI [-0.0309; -0.0029]). Country controls (*p* = .001) showed that in Austria, coercive power was positively related to implicit trust (*b* = .19, *SE* = .37, *p* = .024) whereas it was negatively related to implicit trust in Finland (*b* = -.38, *SE* = .08, *p* <.001) and Hungary (*b* = -.37, *SE* = .09, *p* <.001). However, in the final model, concerning the relationship between implicit trust and tax compliance, the country controls were not significant (all *p’s* > .22).

The second analysis comprised legitimate power as predictive and tax compliance as criterion, whereby reason-based trust, service climate, and voluntary cooperation were the mediators (Figure 2(b)). Country controls (*p* = .001) showed that the positive relationship between legitimate power and reason-based trust varied in strength but not direction (Austria: *b* = .67, *SE* = .06, *p* <.001; Finland: *b* = .90, *SE* = .06, *p* <.001; Hungary: *b* = .90, *SE* = .08, *p* <.001). In the final model, concerning the relationship between reason-based trust and tax compliance country controls were not significant (all *p's* > .25).

The third analysis comprised implicit trust as a predictor and tax compliance intention as criterion, whereby confidence climate and committed cooperation were the mediators (Figure 2(c)). Country controls (*p* <.001) showed that in Finland and Hungary, the relationship between implicit trust and tax compliance is only mediated via committed cooperation (Finland: *b* = .31, *SE* = .06, *p* < .001, Hungary: *b* = .24, *SE* = .06, *p* < .001) but not via the confidence climate (min. *p* = .24). However, in the final overall mediation model, country controls were not significant (all *p's* > .25).

## Discussion

The present paper examined the dynamic between power and trust as outlined in the extended slippery slope framework in three different countries that vary in tax regulations and citizens’ trust. The aim was to shed light on how classical deterrence instruments of audits and fines and alternative approaches of participation, fairness, and trust approaches impact relationship climates, motivation to comply, and intended tax compliance. Therefore, the present paper also informs the broader study of tax policy by highlighting that authorities’ instruments do not impact taxpayers’ compliance in a vacuum but change the relationship climate between tax authorities and taxpayers and the motivation of taxpayers. Thus, the present paper sheds light on complex, underlying psychological mechanisms that enable power and trust to indirectly – versus directly – affect tax compliance. Survey results from taxpayers confirm most assumptions of the extended framework and show that based on data from countries with different tax cultures, general patterns of how tax instruments influence tax compliance can be found. Even if these general patterns vary in a single country such that in one country trust, climates or motivations are more or less affected by tax instruments, the overall relationship and effect on citizens’ perceptions and behaviours is likely very similar among different countries.

Overall counties, coercive power was negatively related to implicit trust and in turn to intended tax compliance. Specifically, this result confirms that coercive powers’ positive impact on tax compliance is undermined if coercive power reduces implicit trust (Gangl et al., 2015). This outcome also might explain previous studies showing weak or no relationship between coercive power and compliance (Ariel, 2012; Hofmann et al., 2014). However, in one country (i.e., Austria) coercive power was positively related to implicit trust. This result indicates as previous studies did, that the negative relationship between coercive power and implicit trust is not stable (c.f. Hofmann et al., 2014, 2017) and may depend on the cultural background and related to that, to the perception of coercive power as safeguard or threat.

In the mediator analysis, results showed that for all countries, legitimate power positively impacted tax compliance intention only via reason-based trust. This result confirms previous studies about the relationship between legitimacy and trust in police studies (Jackson et al. 2012) and tax research (e.g. Gangl et al. 2013; Hartl et al. 2015; Verboon and van Dijke 2011) and again highlights the unconfined positive effect of legitimate power on trust, motivation, and compliance.

Results confirm that the impact of implicit trust on tax compliance intentions is mediated via a perceived confidence climate and committed cooperation. Country characteristics are only marginally relevant for this relationship. These findings extend previous empirical research on the importance of implicit trust for climates and motivations (Gangl et al. 2016). In addition, this result highlights the importance of tax authorities’ instruments that foster implicit trust. Marketing measures (Cyan, Koumpias, and Martinez-Vazquez 2017), the communication of warmth and friendliness (e.g. via a website, telephone hotline, or officers), the use of symbols of legitimacy (e.g. flags, stamps, certifications; Gangl, Torgler, and Kirchler 2016), or highlighting shared values can increase tax motivation as a moral duty and in turn improve tax compliance intentions.

The present research has clear practical implications. Whereas coercive power can have negative side-effects, fostering legitimate power seems to have a positive impact on a large set of indicators such as trust, climate, motivation, and compliance. Thus, tax authorities’ instruments that aim to increase their perceived law-abiding behaviour, professionalism, and expertise; provide clear and transparent information on tax procedures; and foster a positive reputation as a service provider can increase citizens’ trust and cooperation. Tax authorities should also consider ways to increase taxpayers’ implicit trust. Such measures (e.g. building a long-term relationship between a specific tax officer and taxpayer) have a significantly positive influence on compliance.

The present research must be considered in light of specific strengths and limitations. A clear strength of the present research is the examination of taxpayer data from three countries with different legislation and citizens’ levels of trust. Since general assumptions of the eSSF received support among all countries, they can be seen as relatively robust. A limitation of the present research is that only intentions and not real behaviour was assessed. However, intentions are predictors of fraudulent behaviour (Carpenter and Reimers 2005); thus, the present results may have some validity for the field.
